# Visual and skill effects on soccer passing performance, kinematics, and outcome estimations

**DOI:** 10.3389/fpsyg.2015.00198

**Published:** 2015-03-02

**Authors:** Itay Basevitch, Gershon Tenenbaum, William M. Land, Paul Ward

**Affiliations:** ^1^Sport and Exercise Sciences, Anglia Ruskin University, Cambridge, UK; ^2^Educational Psychology and Learning Systems, Florida State University, Tallahassee, FL, USA; ^3^Kinesiology, Health, and Nutrition, University of Texas, San Antonio, TX, USA; ^4^School of Human and Health Sciences, University of Huddersfield, Huddersfield, UK

**Keywords:** action representation, vision, motor performance, error estimation, skill level

## Abstract

The role of visual information and action representations in executing a motor task was examined from a mental representations approach. High-skill (*n* = 20) and low-skill (*n* = 20) soccer players performed a passing task to two targets at distances of 9.14 and 18.29 m, under three visual conditions: normal, occluded, and distorted vision (i.e., +4.0 corrective lenses, a visual acuity of approximately 6/75) without knowledge of results. Following each pass, participants estimated the relative horizontal distance from the target as the ball crossed the target plane. Kinematic data during each pass were also recorded for the shorter distance. Results revealed that performance on the motor task decreased as a function of visual information and task complexity (i.e., distance from target) regardless of skill level. High-skill players performed significantly better than low-skill players on both the actual passing and estimation tasks, at each target distance and visual condition. In addition, kinematic data indicated that high-skill participants were more consistent and had different kinematic movement patterns than low-skill participants. Findings contribute to the understanding of the underlying mechanisms required for successful performance in a self-paced, discrete and closed motor task.

## INTRODUCTION

Visual information plays an important role in the control and production of movement (Robertson and Elliott, 1996a). While visual information is fundamental to movement, how it is utilized to control and coordinate movement is rather ambiguous (Davids et al., 2005). Some findings indicate that as skill level increases, the reliance on online visual information decreases during the execution of a motor task (Abernethy et al., 1994). Underlying this process, contemporary theories on expertise suggest that movement execution is mediated by an internalized mental representation of the action, which alleviates the demands on sensory information required during the execution of movement (Ericsson, 2003; Hodges et al., 2007; Schack and Mechsner, 2006; Schack and Ritter, 2013). Thus, the main purpose of the current study was to delineate the roles of visual information and mental representations in high and low skill participants when performing a motor task.

The action representations of experts are stated to be hierarchically organized containing cognitive motor units, which act to guide the planning and execution of actions (Schack and Mechsner, 2006). In contrast, the representations of novices have been shown to be less hierarchically organized, and not well matched to the biomechanical demands of the task (e.g., Schack and Hackfort, 2007; Bläsing et al., 2009). Analysis of concurrent and retrospective verbal reports in various domains further reveals that skilled performers form rich and detailed representations of performance (e.g., performance goals, monitoring, and alteration; Ericsson and Lehmann, 1996). For example, Gray et al. (2007) examined differences in outcome predictions between expert and novice baseball players using a virtual reality-batting task. Experts predicted more accurately the radial error of ball location, thus highlighting reliance on more elaborate mental representations. Consequently, high-skill participants are better able to estimate outcome results than low-skill participants, who have not yet developed a refined and well-organized action representation.

In principle, this view is consistent with previous motor skill research that suggests that movement planning and preparation can occur prior to initiation of movement (Klapp, 1995). Evidence for preplanned movement control has been provided by deafferentation studies (e.g., Jackson et al., 2000) in which sensory information to the central mechanism were blocked; yet motor tasks could still be performed. Thus, providing support to the notion that movements are organized by central mechanisms, such as motor programs or mental representations in the absence of sensory information (Bard et al., 1999; Hermsdörfer et al., 2008). However, to further capture the underlying mechanisms mediating performance (e.g., action representations and schemas) a more detailed examination of the role of visual information in the production of superior performance is essential (Basevitch, 2009).

Visual information in movement regulation was studied by asking participants to perform a task in the absence of vision (e.g., Robertson and Elliott, 1996a). The general claim was that as individuals increased their level of skill in executing a particular movement pattern they would be less susceptible to interference (i.e., movement disruption) from the removal of vision. While visual-perceptual input has been shown to be an important source of information to regulate action (Caljouw et al., 2004), higher skilled individuals possess more detailed representations of action that are capable of mediating movement execution in the absence of vision. Robertson and Elliott (1996a), for example, asked expert and novice gymnasts to perform movements under three visual conditions: full vision, no vision and displaced vision (15° to the left/right). Expert gymnasts completed the task at the same pace under the vision and no vision conditions. Novices, on-the-other-hand performed the task slower without vision (Basevitch, 2009). The displaced visual condition resulted in the greatest impact on timing for both expert and novice performers.

Consistent with the initial claim, the similarity in coordination pattern executed by the expert gymnasts across visual conditions suggested that their performance was primarily governed via a stored representation of action, and/or by the use of proprioceptive, vestibular, and tactile feedback (Robertson and Elliott, 1996b). However, the disruption to the speed at which the movement was executed, when vision was distorted, also suggests that visual information was used for controlling temporal components of the task. Thus, when visual information was available, information could not be ignored, and was utilized regardless of skill level. However, overall superior performance by expert gymnasts was largely assumed to be controlled by an underlying action representation.

While possessing a detailed action representation may decrease dependence on the visual system, it is evident that visual information still plays a crucial role in movement production, even for expert performers. However, how this information is utilized is still ambiguous (Mann et al., 2007a). Bootsma and van Wieringen (1990) investigated temporal accuracy of elite table tennis players using an attacking forehand drive, in which movement variability measurements were recorded. Findings suggested that temporal variability in movement execution by skilled players was larger during the initial stage of the attacking forehand drive (i.e., onset of forehand stroke) than during the latter stages of the forehand drive prior to and at the moment of ball contact (Basevitch, 2009). Thus, it seems that visual information is important at various temporal points of movement depending on the specific motor task. Unfortunately, in the study outcome measurements (e.g., performance quality) were not reported, making it difficult to conclude that such use of visual information mediated superior, or even successful, performance. Furthermore, in goal-directed movements, a reduction in movement variability, as time-to-contact approaches, is taken to represent the reliance on online visual information by the motor system as it adapts to changes in system constraints (Davids et al., 2008). Therefore, decreases in movement variability have been suggested to reflect the use of online visual information during the execution of movements.

It is important to note that the relationship between performance skill and visual information is moderated by task-type (i.e., interceptive to self-paced). Specifically, findings of a meta-analysis examining perceptual-cognitive expertise in sport provided evidence that gaze behaviors and response measurements (e.g., time and accuracy) were moderated by task type (Mann et al., 2007b). In tasks that adhere to these qualities (i.e., discrete, closed, and self-paced), the environment is relatively static during task performance, thus implying that visual information is less crucial and supporting the mental representations approach. Whereas in relatively open (e.g., table tennis), continuous (e.g., long jump and juggling) and interceptive tasks (e.g., baseball and catching tasks), in which the environment is consistently changing, and there is some amount of unpredictability during performance, visual information, and environmental cues are required for successful performance (Rosenbaum et al., 2006; Basevitch, 2009). Thus, the extent to which online visual information influences performance may be moderated by task type.

Thus, the specific research question addressed in the study was: What role does visual information and mental representations play in performing a discrete, self-paced and closed motor task in high and low skill level soccer players? The underlying theoretical framework that guided the study was the mental representations approach (Ericsson, 2003). Specifically, that performance is mediated by internalized mental representations and that with task specific expertise mental representations are acquired.

In the study we examined the influence of vision (e.g., full, occluded, and distorted vision) on soccer passing performance, movement variability, and error detection capabilities in both high- and low-skilled soccer players. The soccer passing skill is a relatively discrete, closed, and self-paced task and the task environment is relatively static. Thus, skilled performance was assumed to be largely a consequence of representational control. The distorted visual condition further allowed for the verification of the role of action representations in motor control. Specifically, if limited or incorrect visual information (i.e., distorted vision) has little impact on skilled performance, then performance must be mediated by action representations, even when vision is imperfect (Basevitch, 2009). Therefore, high-skill soccer players were expected to perform similarly (i.e., outcome variables—accuracy and consistency) and exhibit comparable coordinative movement patterns (i.e., technique—knee angle and position of supporting foot) and movement variability, across visual conditions.

Conversely, low-skill players, who have not acquired advanced representation, and consequently rely on visual information in performing a motor task, were expected to perform better (i.e., accurately and consistently) in the normal vision condition compared to the distorted and occluded conditions. Furthermore, more movement variability was expected in the distorted and occluded conditions. Finally, it was expected that high-skill players would be better at estimating their level of accuracy in the absence of knowledge of results (Ericsson and Lehmann, 1996), because they are able to access acquired mental representations which helps interpret proprioceptive information (e.g., perceptual movement effects, Schack and Tenenbaum, 2004).

## MATERIALS AND METHODS

### PARTICIPANTS

Forty participants (20 high-skill, and 20 low-skill) were recruited from two universities located in the southern United States. High-skill participants (6 women, 14 men; 18 right-foot dominant, 2 left-foot dominant) ranged from 18 to 39 years of age (*M* = 24.75, SD = 6.95). They were either current or past collegiate soccer players with more than 9 years of soccer experience (*M* = 17.3, SD = 6.42), including 7 years (*M* = 15.7, SD = 6.30) at a competitive level. Low-skill participants (9 women, 11 men; 19 right-foot dominant, 1 left–foot dominant) ranged from 18 to 35 years of age (*M* = 27.55, SD = 3.75). They had played organized soccer for less than a year (*M* = 0.2, SD = 0.41) and had less than 3 years of soccer experience of any type (*M* = 1.6, SD = 1.39). All participants had normal vision.

### PASSING TASK

Participants were asked to pass an official-size soccer ball, by taking one step toward the ball and striking it with the instep of the alternate foot, to a stationary target-mimicking a real player in size and dimension—from one of two different distances: 9.14 and 18.29 m^1^. The task was completed in a natural environment (i.e., outdoors, on a grass field that was maintained constantly to assure relatively similar conditions across players).

### VISUAL CONDITIONS

**FIGURE 1:**
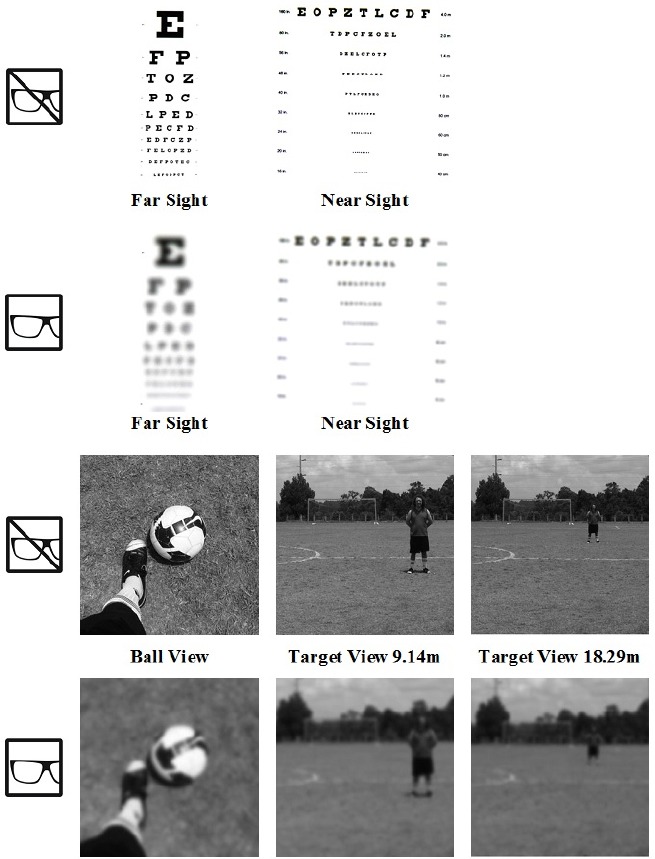
**Simulation of the player’s view with normal and +4.00 corrective lenses of a near/far eye chart and the experimental environment**.

Three visual conditions were used: normal, occluded, and distorted. In the normal condition participants wore large, round eye-glass frames without lenses (Basevitch, 2009). In the occluded condition, participants wore the same frame as in the normal condition with lenses that were completely blacked out to occlude the participants’ vision (and eliminate peripheral information). In the third condition, participants wore the same frame as in the previous two conditions, containing lenses (i.e., +4.00 corrective lenses) that distorted visual acuity^2^ to approximately 6/75, which constitutes legal blindness (Grosvenor, 1996). In this distorted visual condition, participants experienced blurriness and objects appeared larger and closer to mimic shortsightedness (see Figure 1). Previous studies examining the effect of visual acuity on performance (Applegate and Applegate, 1992; Mann et al., 2007a) have used similar methods to degrade visual acuity.

### APPARATUS

Two JVC Everio hard disk camcorders (Model GZ-MG555; JVC America Corp.) were used to videotape performance on the passing task. One camera faced the target and was situated 2.74 m behind and 1.83 m to the right (for left footed participants the camera was situated 1.83 m to the left) of the participant. The second camera, which recorded movement/kinematics at 60 Hz, was focused on the participant, and was situated 9.14 m to the right of the participant (for left footed participants the camera was situated 9.14 m to the left). Analysis was conducted using MaxTRAQ v. 2.08 and MaxMATE v. 3.6 motion capture and analysis software (Innovision Systems, Inc.). The software was used primarily to record outcome measures and kinematic variables of the soccer pass.

#### Demographic questionnaire

Demographic details such as age, gender, years playing soccer (organized and in general), age when first started playing soccer, use of glasses, and dominant (passing) foot were collected (Basevitch, 2009).

#### Perceived outcome form

Following each pass participants were asked to estimate the accuracy of the pass (knowledge of results was not available to the participants—see procedure section for details). Accuracy was operationally defined as the ball’s horizontal distance from the target (in meters) upon crossing the target line (Basevitch, 2009). Participants verbalized whether they perceived the ball hit the target, missed to the left or right of the target, and the estimated distance from the target, if they perceived a miss (e.g., Left, 3 m). The target was the reference point (e.g., 0). A perceived hit was recorded as 0 distance from the target. A perceived miss to the left was recorded as a negative distance (e.g., –3.4 m) relative to the target, and a miss to the right was recorded as a positive distance (e.g., +3.4 m) relative to the target.

### PROCEDURE

Participants were asked to read and sign a consent form, and provide demographic information. They were given six practice trials (e.g., one practice pass for each condition/distance) to become familiar with the task. Each participant then performed a total of 120 trials, split into six blocks. Each block consisted of 20 passes to one of two different distances; 9.14 or 18.29 m, and under one of three different visual conditions; normal, occluded, or distorted. Therefore, each trial block comprised a specific test condition (e.g., 9.14 m-occluded vision). The order of each trial block was counter-balanced across all participants.

For each trial the ball was placed on a marked spot, and the participants positioned and adjusted themselves (Basevitch, 2009). The researcher placed the glasses (depending on the visual condition: frame only, occluded glasses, near-sighted glasses) on the participant, and then prompted the participant to pass the ball to the target (“pass the ball on the ground to the target”). Immediately after contact, a dark opaque screen (dimensions—1.5 × 2 m) was positioned, by the researcher, 1.5 m in front of the participant to prevent outcome feedback (i.e., knowledge of results). This procedure was instituted for the vision and distorted conditions only (in the occluded condition feedback was already blocked). The participants were then asked to estimate the accuracy of their shot. This procedure followed the same routine for all six conditions. Informed consent was obtained prior to data collection and all procedures conformed to APA and institutional ethical review board guidelines.

### ANALYSES

#### Outcome performance

Horizontal distance from the target was used to measure outcome performance. The passing task required the participants to pass the ball on the ground. Vertical distance and depth were not important measurements for this task. With the use of the motion analysis software (MaxTRAQ), balls were marked when they crossed the target line. The markings were then converted to numerical data (with the use of MaxMATE), and absolute distances (i.e., absolute error, AE) from the target were calculated for each pass to measure accuracy. Additionally, to measure performance consistency, variable error (VE) was calculated.

#### Estimated performance

Differences between participants’ estimations and actual performance were calculated. Accuracy (AE) measurements were reported and analyzed.

#### Movement performance

Kinematic measurements were used to examine technique and variability of passing performance (i.e., movement). Using the motion analysis software (MaxTRAQ), key elements of the body (e.g., knee, hip, and ankle of the kicking leg and foot of the supporting leg) were marked during the performance of the task. Specifically, knee angle at the height of the backswing (i.e., angle formed from heel to knee to hip) and relative position of the supporting foot from the ball at contact (i.e., distance from the heel of the foot to the edge of the ball) were the two main variables that were produced and analyzed. Previous research indicated the importance of the two movement variables in soccer type skills such as passing and shooting (Asai et al., 2002). It is important to note that there are several other imperative kinematic variables (e.g., acceleration and velocity of passing foot; Kellis and Katis, 2007). However, because of the nature of the task (i.e., passing task on the ground), instructions (i.e., pass the ball as accurately as you can to the target) and outcome measurements (i.e., accuracy and not power) emphasis was on two variables that are related to accuracy (Orloff et al., 2008). Analysis of kinematic data is presented only for passes in the shorter distance conditions, because the camera recording the kinematic data was only used in the shorter distance.

#### Statistical analyses

A mixed-design ANOVA was used to analyze AE and VE for the actual and estimation data. Skill was the between-participants factor, and distance (from the target) and visual condition were within-participants factors. A mixed-design ANOVA was also used to analyze the mean (i.e., technique) and standard deviation (i.e., variability) of the two kinematic variables, knee angle and position of supporting foot from the ball. Bonferroni *post hoc* pairwise comparisons were performed when required. In addition, partial eta squared (${\text{η}}_{\text{p}}^{\text{2}}$) was used as a measure of effect size. Cohen’s *d* was also used where appropriate.

## RESULTS

This section may be divided by subheadings. Footnotes should not be used and have to be transferred into the main text.

### ACTUAL OUTCOME RESULTS

#### Absolute error

**FIGURE 2:**
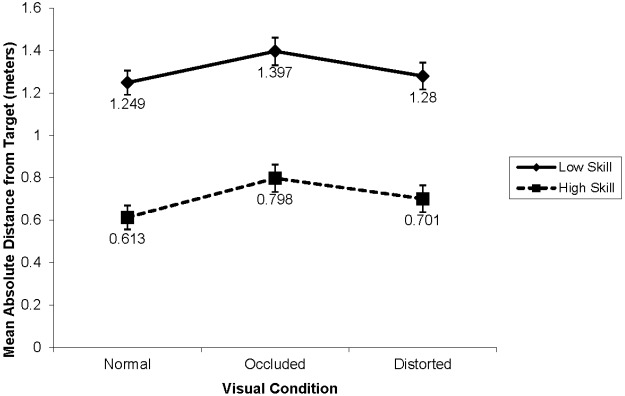
**Mean absolute distance from the target by skill-level and visual conditions (in meters)**.

The analysis of absolute distance from the target revealed a significant main effect for skill, *F*(1,38) = 66.77, *p* < 0.001, ${\text{η}}_{\text{p}}^{\text{2}}$ = 0.64 (see Figure 2). High-skill participants were significantly more accurate (M = 0.70, SD = 0.09) than low-skill participants (M = 1.30, SD = 0.32, d = 2.59). In addition, significant main effects were revealed for distance and visual condition, F(1,38) = 223.20, p < 0.001, ${\text{η}}_{\text{p}}^{\text{2}}$ = 0.86, and F(2,76) = 9.08, p < 0.001, ${\text{η}}_{\text{p}}^{\text{2}}$ = 0.19, respectively. Participants performed with greater accuracy at the shorter (M = 0.582, SD = 0.26) than the longer distance (M = 1.431, SD = 0.56, d = 5.41). Pair-wise comparisons indicated that under the normal visual condition, accuracy was greater than under the occluded condition (p = 0.001; d = 0.55). However, no significant differences resulted between the normal and distorted visual conditions (p = 0.325; d = 0.20), as well as between the occluded and distorted conditions (p = 0.062; d = 0.20).

The only significant interaction was that for Skill × Distance, F(1,38) = 12.61, p < 0.001, ${\text{η}}_{\text{p}}^{\text{2}}$ = 0.25. Distance affected low-skill participants (d = 3.82) more than high-skill participants (d = 2.35). Furthermore, the interaction between skill and visual condition was not significant, F(2,76) = 0.27, p = 0.76, ${\text{η}}_{\text{p}}^{\text{2}}$ = 0.01. Thus, contrary to expectations, visual conditions affected both high- and low-skill participants similarly (see Figure 2).

#### Variable error

**FIGURE 3:**
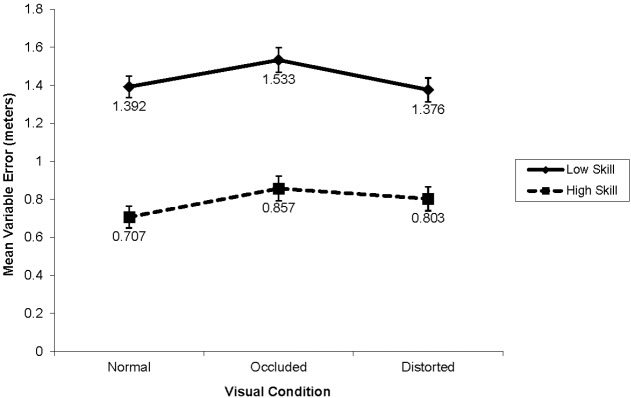
**Mean variable error from the target by skill-level and visual conditions for outcome results (in meters)**.

Significant main effects were observed on variability of outcome performance for skill, *F*(1,38) = 72.37, *p* < 0.001, ${\text{η}}_{\text{p}}^{\text{2}}$ = 0.66, distance, F(1,38) = 54.32, p < 0.001, ${\text{η}}_{\text{p}}^{\text{2}}$ = 0.90, and visual condition, F(2,76) = 5.38, p < 0.01, ${\text{η}}_{\text{p}}^{\text{2}}$ = 0.12 (see Figure 3). High-skill participants were more consistent than low-skill participants (M = 0.789, SD = 0.11 and M = 1.434, SD = 0.32; d = 2.68). Furthermore, consistency decreased as a function of distance; VE was smaller at 9.14 m compared to 18.29 m from the target (M = 0.636, SD = 0.26 and M = 1.587, SD = 0.57, d = 5.11 respectively). Pair-wise comparisons revealed that consistency was higher in the normal visual condition than in the occluded condition (p = 0.005; d = 0.50). Similarly to AE, the distorted visual condition was not significantly different from both the occluded and normal conditions (p = 0.153; d = 0.34, and p = 1.000; d = 0.13, respectively).

A Skill × Distance interaction was revealed for VE, F(1,38) = 16.86, p < 0.001, ${\text{η}}_{\text{p}}^{\text{2}}$ = 0.31. Target distance affected the consistency of low skill participants (d = 4.43) significantly more than distance affected the consistency of high-skill participants (d = 2.84). The skill by visual condition interaction was not significant, F(2,76) = 0.91, p = 0.405, ${\text{η}}_{\text{p}}^{\text{2}}$ = 0.021.

### ACTUAL—ESTIMATION DIFFERENCES

#### Absolute error

Main effects for skill were obtained for AE, *F*(1,38) = 44.742, *p* < 0.001, ${\text{η}}_{\text{p}}^{\text{2}}$ = 0.54. High-skill participants were more accurate in estimating the final ball location (i.e., AE; M = 0.469, SD = 0.12) than the low-skill participants (M = 0.847, SD = 0.22, d = 2.10). Furthermore, a Skill × Distance interaction was obtained for AE, F(1,38) = 54.32, p < 0.001, ${\text{η}}_{\text{p}}^{\text{2}}$ = 0.90. Both high- and low-skill players estimated the ball location better at the shorter distance. However, the negative effect of distance on performance estimation was significantly greater for the low-skill group (d = 2.75) than for the high-skill group (d = 1.56).

**FIGURE 4:**
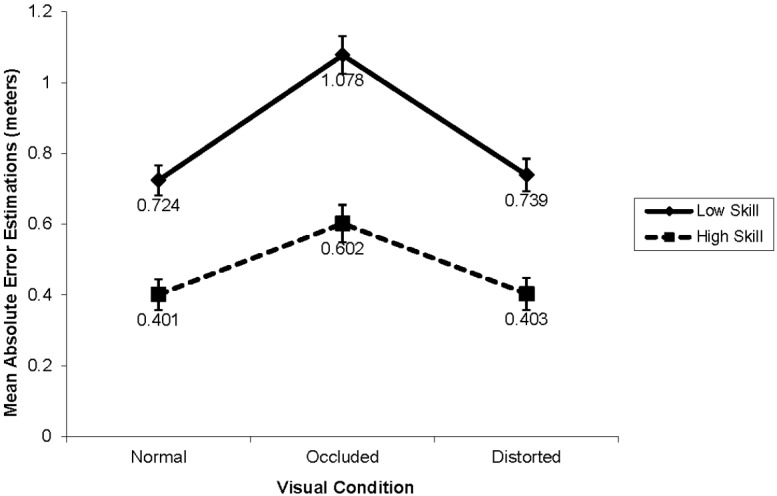
**Mean AE of estimations by skill-level and visual conditions**.

The Skill-level × Visual condition interaction was not significant. However, it approached significance (i.e., p = 0.072, see Figure 4). Further examination of standardized mean differences (d, Cohen, 1988) revealed that the difference in estimation accuracy between high-skill and low-skill participants was larger in the occluded condition (i.e., d = 2.77) than in the normal condition (i.e., d = 2.58), and distorted condition (i.e., d = 1.97).

### KINEMATIC RESULTS

#### Variability (SD)

Analysis revealed a significant main effect for skill-level for peak knee angle at backswing, *F*(1,38) = 5.884, *p* < 0.05, ${\text{η}}_{\text{p}}^{\text{2}}$ = 0.13, and for relative position of supporting foot at contact, F(1,38) = 7.755, p < 0.01, ${\text{η}}_{\text{p}}^{\text{2}}$ = 0.17. High-skill participants (knee angle: M = 6.223, SD = 1.77 and supporting foot position: M = 0.083, SD = 0.03) were more consistent than low-skill participants (knee angle: M = 9.179, SD = 5.16, d = 0.79 and supporting foot position: M = 0.110, SD = 0.03, d = 0.73) across the visual conditions. There were no significant main effect for visual condition and no Vision × Group interaction.

#### Technique (M)

A significant main effect for skill-level for relative position of the supporting foot at contact was revealed, *F*(1,38) = 4.225, *p* < 0.05, ${\text{η}}_{\text{p}}^{\text{2}}$ = 0.10. High-skill participants positioned their supporting foot relatively closer to the ball (M = 0.369, SD = 0.12) than low-skill participants (M = 0.506, SD = 0.27, d = 0.30). In addition, a significant main effect was revealed for visual condition in relative position of supporting foot from the ball at contact, F(2,76) = 27.99, p < 0.001, ${\text{η}}_{\text{p}}^{\text{2}}$ = 0.424. Participants positioned their supporting foot relatively farther from the ball in the distorted condition compared to both the normal and occluded visual conditions (M = 0.511, SD = 0.23; M = 0.391, SD = 0.20; and M = 0.411, SD = 0.25 respectively). There were no significant main or interaction effects for peak knee angle at backswing.

## DISCUSSION

The aim of the study was to delineate the role of visual information and mental representations in performing a discrete self-paced motor task. Data relating to action representations during performance (i.e., outcome estimations) were collected in addition to behavioral data (i.e., outcome performance and kinematic movement) of low- and high-skill soccer players.

### OUTCOME PERFORMANCE

As expected, high-skill players performed the passes significantly more accurate and consistent in all three visual conditions. Both high- and low-skill players performed better (i.e., accurately and consistently) at the shorter distance, in line with previous findings (Aksamit and Husak, 1983; Wannebo and Reeve, 1984; Sutter, 2007). However, in the current study, distance did not affect high-skill players as much; findings aligned with Guadagnoli and Lee’s (2004) review, explaining that as task difficulty increases performance of low skill individuals’ declines rapidly and at relatively low levels of nominal difficulty.

More importantly, it seems as though visual information affected both high- and low-skill players similarly, as evident by non-significant Skill × Vision interactions. These results indicate that both skill level groups utilize visual information similarly, to some degree, when performing a motor skill even in a relatively self-paced, discrete, and closed task such as a set-play soccer pass. Moreover, it seems mental representations did not have a significant role in determining the outcome performance on the task for both high and low-skill players. A plausible explanation to the findings could be that the high-skill players have not acquired advanced mental representations, as they are not experts in their domain (Ericsson, 2003).

Similar findings were found in a study examining golf performance (e.g., outcome performance only; kinematic and cognitive measures were not examined) of low- and high-skill players in a putting task (Wannebo and Reeve, 1984) where participants were required to perform a putt to a target at various distances (i.e., 5 and 15 ft) under various visual conditions (i.e., normal, blindfolded, and irrelevant cues). Results revealed significant main effects for skill-level and visual conditions, however there were no significant interactions between the two factors, similar to the current study’s results. Thus, the authors concluded that visual information is important in golf putting for both low- and high-skill level groups (Wannebo and Reeve, 1984). Future research should examine distorting/occluding specific cues (e.g., the ball, target or limbs), to gain a better understanding of the specific environmental information that is necessary for successful performance (Elliott, 1988). Furthermore, a limitation of the study was the relatively small sample size and large SD resulting in low statistical power in some of the analyses. Future studies should include larger samples and make sure that participants in each skill level group are less diverse (e.g., similar amount of experience, same level of performance).

The current results support the notion that some visual information is better than none, and that perhaps even if visual information is not accurate, and is altered, the perceptual-motor system is able to compensate and adjust (Mann et al., 2007a). Nevertheless, looking at the descriptive data, it seems that performance under the distorted condition was placed in between the other two visual conditions; better than the occluded condition and worse than the normal condition (Basevitch, 2009). Indeed, in a study examining the effect of visual acuity on performance in an interceptive task (i.e., cricket batting), findings indicated that only a distortion comparable to that of legal blindness resulted in a significant decrement of performance (Mann et al., 2007a). Distortion levels that were less extreme did not result in significant performance decrements. In a similar study, Applegate and Applegate (1992) explored the effect of visual acuity on static shooting performance in basketball. As with the current study, even visual acuity of 6/75 did not significantly reduce performance. Hence, if for an interceptive task, such as cricket batting, severe distortion is needed to significantly affect performance, and in a self-paced task such as a set-shot in basketball, similar distortions have little effect on performance, then, in a set-play soccer pass, in which vision is potentially less important, a similar distortion might not significantly affect performance, as the findings of the current study indicate. Thus, the findings support the notion that outcome performance is not affected by the quality of vision in a task that is discrete, closed and self-placed such as the soccer task in the current study.

### ACTUAL—ESTIMATION DIFFERENCES

To further capture the influence of underlying mental representations, the differences between participants’ outcome estimation and actual performance were examined. It was expected that high-skill players who have developed refined representations with practice, should estimate outcome results more accurately than low-skill participants when feedback is limited, especially under occluded conditions where the feedback available is minimal (Gray et al., 2007). Findings from the study supported this notion in that high-skill players estimated final ball location more accurately than low-skill participants. Additionally, even though the Skill × Vision interaction was not significant, further examination revealed that the greatest estimation differences (i.e., *d* = 2.77) between the two skill-level groups occurred in the occluded condition. In the occluded condition reliance on action representations is presumed to be stronger, because visual information is not available (Basevitch, 2009). Thus, the findings give further support to the notion that representations mediate performance, and that high-skill performers acquire more refined internal representations, which allow them to achieve more accurate and consistent outcomes (Ford et al., 2007).

### KINEMATIC PERFORMANCE

To examine the role visual information plays in movement production both outcome performance and movement kinematics were analyzed integratively in line with previous research (Bennett and Davids, 1995; Ford et al., 2006). High-skill players were more consistent than low-skill players irrespective of visual condition in both peak knee angle at backswing, and in position of supporting foot at contact. Additionally, contrary to expectations, visual information failed to affect low-skill players’ movement variability for both kinematic variables. It is likely that players compensated for the reduced visual information by attending to other environmental cues, utilizing additional sensory systems (Ford et al., 2007). For example, participants could have compensated for the reduction of visual information by attending to the auditory information available, such as listening to the sound of the ball as it bounces on the grass, or being aware of their own movement (i.e., proprioception; Robertson and Elliott, 1996b). Therefore, it is possible that players used on-going sensory information as feedback, adjusting their movement accordingly (Ford et al., 2006). Future studies should examine movement variability with the reduction or elimination of other sources of information (e.g., audio), in addition to visual information.

Finally, high-skill players employed a different movement pattern only in the positioning of their supporting foot at contact. To this extent, high-skill players positioned their foot relative to the ball differently depending on visual condition. While vision affected the positioning of the supporting foot in the skilled performers, it did not affect peak knee angle variability during the back swing. Given that visual occlusion did not influence the degree of variability during the movement suggests that vision did not play a primary role in the online control of skill execution. However, differences in foot placement and outcome performance related to visual condition suggest that visual information was important, but primarily for the preplanning of the movement. The kinematics of the movement did not change, only the parameterization of the overall movement (e.g., lengthening or shortening the run to the ball, thus influence foot positioning). Similar findings have been found by Land et al. (2013) who show that changes to overall movement patterns reflect offline uses of visual information prior to movement execution rather than online visual control. Such findings suggest that vision is important for the performance of the soccer kick in so far that it impacts movement planning and preparation, but not the extent to which it impacts the unfolding and execution of the movement via online regulation.

## CONCLUSION

Although previous studies examined the role of vision on performance, few, if any, have included outcome, movement, and cognitive variables in the same examination. The importance of including all relevant components of the performance process is essential for capturing the mediating processes leading to successful performance. Results from the present study indicate that both mental representations and vision mediate performance. Online visual information is important in producing skilled movements even for high-skill players. However, future studies should include expert level players who have acquired task-specific action representations (i.e., schema). Finally, examining players’ estimations of final ball outcome indicated that the high-skill participants developed action representations that enabled performing at a relatively high level when visual information was not optimal. More research must capture the role of other sensations on motor performance and motor variability in conditions which vary in sensation deprivation. In addition, further research is needed to examine the relationship among the various components (e.g., kinematics, mental representations) at different levels (e.g., motor, cognitive) in producing successful performance.

### Conflict of Interest Statement

The authors declare that the research was conducted in the absence of any commercial or financial relationships that could be construed as a potential conflict of interest.

## Footnotes

1 Distances were selected as less and more difficult after high- and low-skill players pilot tested passing to multiple distances between 5 and 30 m. This was to assure a reasonable level of task difficulty, and to observe differences in performance between the two distances.

2 Six participants were randomly asked to take a visual acuity exam using the Snellen chart wearing the distorted vision glasses. They all had a visual acuity of approximately 6/75.
